# 

**Published:** 1897-09-04

**Authors:** 


### EARLY EDITION.

**Notices of Births, Deaths, and Marriages** are inserted in this column at a charge of 2s. 6d. for an announcement not exceeding four lines.

### NOTICE TO CORRESPONDENTS.

All MS., Letters, Books for Review, and other matters intended for the Editor should be addressed The Editor, "The Hospital," The Hospital Building, 28 & 29, Southampton Street, Strand, W.C.

The Editor cannot undertake to return rejected MS., even when accompanied by stamped directed envelope.

**Notice to Advertisers and Subscribers.**

All Advertisements, Orders for Copies of The Hospital, and Business Communications should be addressed to **THE MANAGER, (Not to The Editor),** The Hospital, The Hospital Building, 28 & 29, Southampton Street, Strand, London, W.C.

The Cost of Subscription, post free, is as follows:—Per week, 2½d.; per quarter, 2s. 8d.; per half-year, 5s. 3d.; per annum, 10s. 6d. Foreign:—Per annum, 15s. 2d.; payable, in all cases, in advance.

### The Student of Medicine.

### ROYAL COLLEGE OF PHYSICIANS AND SURGEONS, EDINBURGH.

The following questions were set in the examination for the triple qualification :—

Elementary Anatomy—Five Years' Course.—(All the questions to be answered.)

1. Describe the outer wall of a nasal fossa.
2. Name in order the bones of the carpus, and give the attachment to bones of the anterior and the posterior annular ligaments at the wrist.
3. Describe the origin and course of the musculo-spiral nerve. Name the muscles directly supplied by it.
4. Describe the external lateral ligament of the ankle-joint.

Anatomy.—Five Years' Course.—(All the questions to be answered.)

1. Describe the anterior annular ligament of the carpus and name the structures which pass beneath and over it.
2. Describe the tunica vaginalis and tunica albuginea of the testis.
3. Describe a normal obturator artery, and state the probable origin of the artery when abnormal.
4. Describe a lateral ventricle of the brain, and name in order the structures which are seen on its floor.

Materia Medica—Four Years' Course.—(All the questions to be answered.)

1. Describe fully the physical characters of chloroform and the tests for its purity? What official preparations contain chloroform? Mention the proportion present in each, and the doses of such as are for internal use.
2. What ingredients enter into the composition of (*a*) compound tincture of camphor, (*b*) compound powder of liquorice, (*c*) compound mixture of senna, (*d*) compound colocynth pill? N.B.—In each case the names of the ingredients to be given in unabbreviated Latin.
3. What is the botanical source of (*a*) Elaterium? What is its active principle and the dose of elaterium? (*b*) Digitalis? Name its liquid preparations and their doses. (*c*) Catechu? Name its official preparations.
4. Describe the appearance of (*a*) Tartarated iron, (*b*) citrate of iron and quinine, (*c*) citrate of iron and ammonia. State what you know of their solubility, and mention the dose of each.

Physiology.—(All the questions to be answered.)

1. The weight of a healthy man is constant within small limits of variation. Describe the processes (*a*) of loss, (*b*) of gain, by which the equilibrium is maintained.
2. What conditions are necessary for the coagulation of the blood (*a*) within the vessels, (*b*) when withdrawn from the vessels?
3. What physiological purposes are served by the elasticity of (*a*) the crystalline lens; (*b*) the walls of the air vesicles of the lung; (*c*) the arteries?
4. Describe the microscopic structure of skin, and the method of making a preparation to show it.

Advanced Anatomy.—Four Years' Course.—(All the questions to be answered.)

1. Describe the tympanic cavity and its contents.
2. Describe the position, relations, and blood supply of the thyroid body.
3. Describe the biliary passages within and beyond the liver.
4. Describe the arterial circulation at the base of the brain.

